# Complex genetic architecture in severe hypobetalipoproteinemia

**DOI:** 10.1186/s12944-018-0680-1

**Published:** 2018-03-14

**Authors:** Linda R. Wang, Adam D. McIntyre, Robert A. Hegele

**Affiliations:** Department of Medicine and Robarts Research Institute, Schulich School of Medicine and Dentistry, Western University, 4288A - 1151 Richmond Street North, London, ON N6A 5B7 Canada

**Keywords:** Hypobetalipoproteinemia, Abetalipoproteinemia, DNA mutations, DNA sequencing, Oligogenic trait, Complex trait, Acanthocytosis

## Abstract

**Background:**

Abetalipoproteinemia and homozygous hypobetalipoproteinemia are classical Mendelian autosomal recessive and co-dominant conditions, respectively, which are phenotypically similar and are usually caused by bi-allelic mutations in *MTTP* and *APOB* genes, respectively. Instances of more complex patterns of genomic variants resulting in this distinct phenotype have not been reported.

**Methods:**

A 43 year-old male had a longstanding severe deficiency of apolipoprotein (apo) B-containing lipoproteins and circulating fat soluble vitamins consistent with either abetalipoproteinemia or homozygous familial hypobetalipoproteinemia (FHBL). He also had acanthocytosis, a long term history of fat malabsorption, and mild retinopathy, but was free from coagulopathy, myopathy and neuropathy. He had taken high dose oral fat soluble vitamins since childhood.

**Results:**

Targeted next generation DNA sequencing revealed several rare heterozygous missense variants in both *MTTP* and *APOB* genes known or predicted to be deleterious, in addition to a novel heterozygous missense variant in *SAR1B*, which encodes the gene causing chylomicron retention disease. Evaluation of first degree relatives with mild FHBL clarified the segregation of variants.

**Conclusions:**

The proband’s characteristic phenotype likely resulted from an oligogenic interaction involving multiple rare variants in *MTTP* and *APOB*, and related genes, each of which individually was associated with a milder or minimal clinical and biochemical phenotype.

## Background

Abetalipoproteinemia (ABL; OMIM 200100) and familial hypobetalipoproteinemia (FHBL; OMIM 615558) are rare genetic disorders affecting packaging and transport of apolipoprotein (apo) B-containing lipoprotein particles, which are essential for the absorption and trafficking of cholesterol and triglycerides [1]. ABL is a classic autosomal recessive disorder in which the affected homozygote has undetectable levels of low-density lipoprotein (LDL) cholesterol (C) and other apo B-containing lipoproteins. Untreated ABL patients develop consequences of fat-soluble vitamin deficiency including atypical retinitis pigmentosa, progressive spinocerebellar degeneration, coagulopathy and osteomalacia [2]. Acanthocytosis on peripheral blood film is a hallmark of ABL. Heterozygous parents of ABL patients have normal lipid profiles and no systemic complications [3]. Homozygous FHBL patients are clinically indistinguishable from ABL, except that their heterozygous parents have low but detectable LDL-C levels and protection from cardiovascular disease [4], consistent with autosomal codominant inheritance. We report an unusual presentation of an adult with either ABL or homozygous FHBL phenotype, but a complex genotype which was not consistent with the typical form of either disorder.

### Subjects and methods

#### Proband and family

A 43-year-old man (subject II-2) was referred for molecular genetic diagnosis of longstanding hypolipidemia. At age one, he presented with chronic diarrhea and failure to thrive. Investigations revealed acanthocytosis on peripheral blood film, unmeasurable LDL-C, and severe fat soluble vitamin deficiencies. He was diagnosed with ABL, treated with high dose oral vitamins A, D, E, and K, and was advised to avoid fatty foods. This led to symptomatic improvement. He was lost to follow-up but adhered to treatment through adulthood, and long sought an accurate diagnosis of his condition.

His medical history includes evidence of retinopathy and glaucoma, mild anemia, osteoarthritis, hydrocele repair and sports-related finger fractures. He was taking oral vitamin A 30,000 IU, vitamin E 3200 IU, vitamin D 2000 IU, vitamin B12 1200 mcg, and travoprost ophthalmic drops daily, and vitamin K 5 mg intramuscular weekly. He endorsed chronic blurry vision, nyctalopia, and mild coordination issues. He was unaffected by numbness, weakness, paresthesiae, gait ataxia, major fractures, or bleeding, and has enjoyed a conventional home and occupational life without impairments.

His father is of Jamaican descent, and mother (now deceased) was from Nova Scotia, Canada, of unspecified African ancestry. His mother underwent coronary artery bypass grafting in her early forties. His brother had type 1 diabetes complicated by cardiac ischemia at age 41, and passed away in his forties. His father and 13-year old daughter are well.

On examination, his weight, height, blood pressure and heart rate were 73.2 kg, 165 cm, 140/85 mmHg and 60/min, respectively. He had bilateral arcus cornealis and atypical retinitis pigmentosa. His cardiovascular, respiratory, and abdominal examinations were unremarkable. He had no xanthomas or xanthelasma. Neurological examination demonstrated normal power and cranial nerves, symmetrical 1+ and 2+ reflexes in the upper and lower extremities, respectively.

**Fig. 1:**
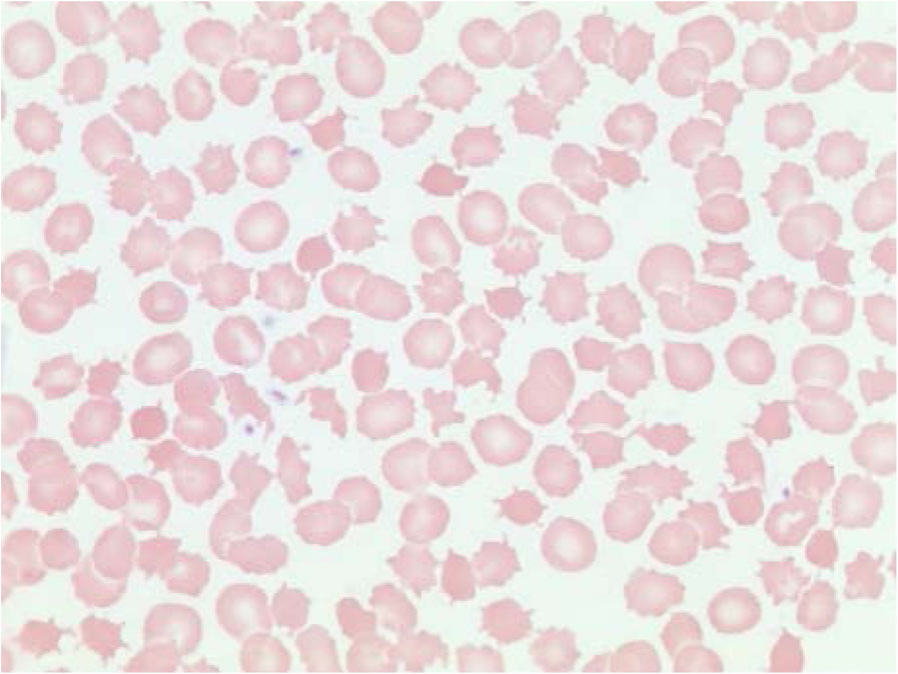
Proband’s peripheral blood film demonstrating acanthocytosis

**Fig. 2:**
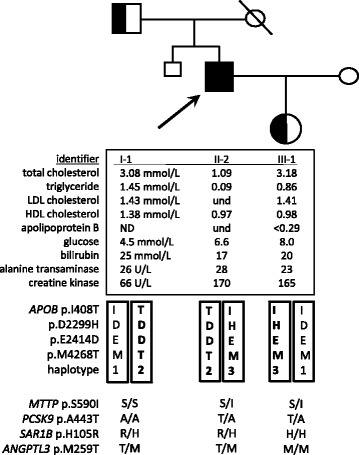
Pedigree showing nuclear family members studied, with selected phenotypes and inheritance of rare mutations identified. Proband indicated by arrow. Abbreviations: as in Table 1, plus LDL, low-density lipoprotein; HDL, high-density lipoprotein; ND, not determined; und, undetectable; U/L, units per litre. Reference ranges for alanine transaminase and creatine kinase are < 42 U/L and < 145 U/L, respectively. Extended haplotypes for the *APOB* locus are shown; chromosomal phase was determined unequivocally and 3 distinct haplotypes (arbitrarily designated 1-3) are shown. APOB haplotypes are indicated in boxes; the likely pathogenic haplotypes are shaded grey. Also, genotypes for variants in Table 1 for *MTTP*, *PCSK9*, *SAR1B* and *ANGPTL3* genes are shown

Laboratory investigations revealed hemoglobin of 134 g/L, platelet count of 147 X 10^5^/L, and a white blood cell count of 4.7 X 10^5^/L. His peripheral blood film showed acanthocytosis with spherocytes, polychromasia and burr cells (Fig. 1). Selected laboratory values are shown in Fig. 2. Notably, he had extremely low levels of total cholesterol, triglycerides, and lipid-soluble vitamins, with undetectable LDL-C. Carotid intima media thickness was at 50th percentile for age and sex. His ECG showed sinus bradycardia. Abdominal ultrasound was negative for hepatic steatosis.

#### DNA analysis

Targeted next-generation sequencing of genomic DNA comprised all known dyslipidemia genes, including *MTTP* (ABL gene), *APOB* (FHBL gene), *PCSK9* and *SAR1B* (chylomicron retention disease gene) [5, 6]. VarSeq software prioritized possible or likely pathogenic rare variants of using the Online Mendelian Inheritance in Man database (https://www.ncbi.nlm.nih.gov/omim/). Prediction algorithms such as the Combined Annotation Dependent Depletion (CADD; 7; http://cadd.gs.washington.edu/); Sorting Intolerant From Tolerant (SIFT; 8; http://sift.jcvi.org/); and Polymorphism Phenotyping tool version 2 (PolyPhen-2; 9; http://genetics.bwh.harvard.edu/pph2/). Minor allele frequencies were compared against those cited for African subpopulations in the Exome Aggregation Consortium database (ExAC; 10; http://exac.broadinstitute.org/). All rare variants were confirmed by Sanger sequencing. Segregation of variants across three generations was demonstrated by genotyping family members using Sanger sequencing.

## Results

While a single homozygous mutation in the *APOB* gene was expected, remarkably 8 heterozygous rare and potentially deleterious mutations were identified in the proband’s genome: four in the *APOB* gene (p.I408T, p.D2299H, p.E2414D and p.M4268T), and one each in *MTTP* (p.S590I), *PCSK9* (p.A443T), *SAR1B* (p.H105R) and *ANGPTL3* (p.M259T) genes. The evidence for dysfunction was strongest for the *MTTP* p.S590I, which in the homozygous state had been reported in two ABL subjects [7, 8] and completely lacks both triglyceride and phospholipid transfer activities in vitro [9]. However, simple heterozygotes had normal plasma lipid profiles, suggesting that *trans-*interacting variants at other loci might be contributing to the proband’s severe phenotype.

The proband’s 82 year-old father (subject I-1) and 13 year-old daughter (subject III-1) were studied to clarify inheritance of lipid phenotype and DNA variants, including the chromosomal phase of the *APOB* variants. Both were asymptomatic with no history of cardiovascular, gastrointestinal, ophthalmological, hepatic, neurological, or hematological problems. The LDL-C level in both the proband’s father and daughter was <5th percentile for age and sex (Fig. 2), consistent with heterozygous FHBL in both. We next focused on the segregation of the four rare *APOB* variants identified in the proband to construct unambiguous haplotypes (Fig. 2).

The reference *APOB* haplotype contained I408, D2299, E2414 and M4268; we called this “haplotype 1”. The proband’s father and daughter were each heterozygous for haplotype 1, which we presumed to be phenotypically neutral. The daughter’s second allele contained only one *APOB* variant, namely H2299, which had the strongest bioinformatic prediction of deleterious effect with concordance across all three software tools (Table 1). We called this *APOB* “haplotype 3”; she inherited this allele from her father the proband, who was also a heterozygote. The proband’s father’s second *APOB* allele contained three rare variants, namely T408, D2414 and T4268 (Fig. 2). Bioinformatic analysis of each variant individually showed less consistent predictions of deleterious effect across the three software programs (Table 1). We called this *APOB* “haplotype 2”, and noted that the proband, like his father, was a heterozygote. These three linked variants might act synergistically resulting in a greater functional compromise than would be predicted for each variant individually; current software algorithms cannot assess this possibility.

**Table 1:** Heterozygous rare variants detected in atypical hypobetalipoproteinemia proband

gene/chr	exon	cDNA change	amino acid	ExAC MAF(A)	CADD	SIFT	PolyPhen-2	comments
*APOB*/2p24	10	c.T1223C	p.I408T	0.0268	15.7	0.211	0.065	uncommon missense variant; possibly pathogenic [17]
	26	c.G6895C	p.D2299H	0.0215	19.6	0.062	0.98	uncommon missense variant; possibly pathogenic [17]
	26	c.A7242C	p.E2414D	0.0076	11	0.405	0.007	uncommon missense variant; unlikely pathogenic
	29	c.T12803C	p.M4268T	0.0076	0.001	0.485	0	uncommon missense variant; unlikely pathogenic [17]
*MTTP*/4q23	13	c.G1769T	p.S590I	NP	28.5	0.034	0.996	very rare proven dysfunctional variant [7, 9]
*PCSK9*/1p32	8	c.G1327A	p.A443T	0.0981	9.68	0.534	0.004	variant of unknown significance
*SAR1B*/5q31	5	c.A314G	p.H105R	NP	9.84	1	0	variant of unknown significance
*ANGPTL3*/1p31	4	c.T776C	p.M259T	0.0542	3.46	0.084	0.001	variant of unknown significance

*Abbreviations: chr* Chromosomal locus, *cDNA* Coding DNA sequence, *APOB* Gene encoding apolipoprotein B, *MTTP* Gene encoding microsomal triglyceride transfer protein, *PCSK9*, Gene encoding proprotein convertase subtilisin kexin 9, *SAR1B* Gene encoding *S. cerevisiae* homolog B (chylomicron retention disease gene), *ANGPTL3* Gene encoding angiopoietin-like protein 3, *ExAC* Exome aggregation consortium [18] (url: http://exac.broadinstitute.org/), *MAF(A)* Minor allele frequency in African populations, *NP* Not present in database, *CADD* Combined annotation dependent depletion algorithm [5] (url: http://cadd.gs.washington.edu/), *SIFT* Sorting intolerant from tolerant algorithm [6] (url: http://sift.jcvi.org/), *PolyPhen-2* Polymorphism phenotyping tool version 2 [19] (url: http://genetics.bwh.harvard.edu/pph2/)

*Explanation of predictive functional scores:* A CADD score > 20 is predicted to be in the top 1% of most deleterious substitutions within the human genome. A CADD score from 10 to 20 is predicted to be in the top 10% of most deleterious substitutions within the human genome. A SIFT score ≤ 0.05 is considered to be ‘deleterious’; while a score ≥ 0.05 is considered to be ‘tolerated’. A PolyPhen-2 score of 0.957 to 1.0 is considered to be ‘probably damaging’; of 0.454 to 0.956 is considered to be ‘possibly damaging’ and of 0.0 to 0.453 is considered to be ‘benign’

Thus, the proband was a compound heterozygote for two rare *APOB* alleles defined by haplotypes 2 and 3. It is feasible that each of these rare *APOB* haplotypes encodes a protein variant with compromised function. The co-segregation of the heterozygous *APOB* alleles with the abnormal lipid phenotypes across the three generations is consistent with severe compound heterozygous FHBL in the proband and simple heterozygous FHBL in his father and daughter. However, it is unclear how the heterozygous *MTTP* p.S590I variant might interact to further affect phenotypes.

Three other heterozygous variants in LDL-C-lowering genes were identified in the proband (Fig. 2), namely *PCSK9* p.A443T, *SAR1B* p.H105R and *ANGPTL3* p.M259T. However, bioinformatic evidence favoring pathogenicity in any of these is marginal (Table 1); it would be most prudent to refer to these as “variants of unknown significance”, although a possible small contributory role for any of these variants in the proband’s severe phenotype cannot be assessed with current tools.

## Discussion

We report a man with longstanding severe hypolipidemia, consistent with ABL or homozygous FHBL, whose first degree relatives each had less severe reductions in apo B-containing lipoproteins, consistent with heterozygous FHBL. Eight heterozygous rare and potentially deleterious missense mutations were identified in the proband’s genome: four in the *APOB* gene, and one each in *MTTP*, *PCSK9*, *SAR1B* and *ANGPTL3* genes. Co-segregation of two rare *APOB* gene haplotypes with depressed LDL-C across three generations was consistent with compound heterozygous FHBL. But the proband was also heterozygous for the proven dysfunctional *MTTP* p.S590I variant, which completely lacks both triglyceride and phospholipid transfer activities in vitro [9]. Additional rare variants in *PCSK9*, *SAR1B* and *ANGPTL3* might have had further additive effects. The presence of so many rare and potentially contributory variants to an extremely low LDL-C and fat soluble vitamin deficiency is unique to date, but may be seen increasingly with routine clinical application of next-generation sequencing.

The differential diagnosis for concurrent undetectable levels of LDL-C, triglycerides, and apo B is limited to ABL and homozygous FHBL [1]. Both rare genetic disorders are characterized by compromised synthesis and secretion of apo B-containing lipoproteins, which are necessary for the absorption of lipids from the gastrointestinal tract. Patients present in infancy with steatorrhea from fat malabsorption and failure to thrive. Without intervention, patients develop severe sequalae of lipid-soluble vitamin deficiency including retinal degeneration, cerebellar ataxia, loss of proprioception, coagulopathy, and osteomalacia. The neurological impairments may gradually become debilitating and result in immobility [1]. Hepatic steatosis and elevated transaminase levels are not uncommon, seen more in HHBL than ABL [10].

ABL and homozygous FHBL are virtually clinically indistinguishable and are recognized based on lipid profile (e.g. LDL-C < 0.1 mmol/L, TG < 0.2 mmol/L, and apo B < 0.1 g/L), peripheral acanthocytosis, and symptoms of fat malabsorption [1]. Definitive diagnosis relies on DNA sequencing to detect the causative variants. ABL results from more than 30 reported homozygous mutations in the *MTTP* gene on chromosome 4q22-24, which codes for microsomal triglyceride transfer protein (MTP). MTP is required for triglyceride transfer onto nascent apo B in the endoplasmic reticulum during the assembly of chylomicrons and VLDL in enterocytes and hepatocytes, respectively [11]. Obligate heterozygous parents of ABL patients have normal lipids, consistent with autosomal recessive inheritance. In contrast, FHBL results from mutations in in the *APOB* gene on chromosome 2p24, which encodes apo B, a necessary component of all apo B-containing particles including LDL, VLDL, and chylomicrons. Homozygous FHBL patients are virtually indistinguishable from ABL patients, but heterozygotes have half-normal levels of LDL-C, consistent with autosomal codominant inheritance [1].

Our proband’s phenotype comprising severe fat soluble vitamin deficiencies, acanthocytosis, undetectable LDL-C and TG, and history of malabsorption pointed to either ABL or homozygous FHBL; that 8 rare and potentially deleterious mutations in candidate genes were discovered presented an interesting diagnostic challenge. Careful genotype analysis was crucial to elucidating the causal contributions of his mutations; with multigenerational participants it was possible to create extended haplotypes of the *APOB* locus and to follow the co-segregation of the mutations and phenotypes. We found that our proband had two distinct variant *APOB* alleles, each characterized by at least one missense variant at one of four sites at the *APOB* gene. Observing a less severe hypolipidemia in each first degree relative with one variant allele supported the diagnosis of compound heterozygous FHBL. Individuals of African ancestry with all four *APOB* variants are exceptionally rare, as is a compound heterozygous genotype accounting for this clinical constellation; a similar case of compound heterozygous FHBL was reported earlier [12].

We further considered the role of his mutations in *ANGPTL3, SAR1B,* and *PCSK9*. Loss of function variants in these genes have been implicated in disorders characterized by extremely low LDL-C, but are not independently consistent with the clinical phenotype or inheritance pattern in this nuclear family. Homozygous or compound heterozygous loss-of-function *ANGPTL3* mutations cause familial combined hypolipidemia [13]; *SAR1B* mutations lead to the autosomal recessive chylomicron retention disease (OMIM #246700) with severe hypocholesterolemia and fat malabsorption, but normal TG; and loss-of-function mutations in *PCSK9* have been famously associated with depressed serum LDL-C, but not to the degree observed here. The multigenerational co-segregation (in particular, comparing I-1 and II-2) also shows *PCSK9* mutation to be insufficient to explain the phenotypic differences. It is conceivable, however, that these other rare heterozygous mutations may contribute in a polygenic manner to the severe phenotype seen in this proband II-1. A patient with so many concurrent rare variants in candidate genes for low LDL-C has not yet been reported. Definitively establishing true causality would require extensive in vitro experiments. Nevertheless, cases like this do provide more evidence which may guide such experimentation.

This case also highlights the importance of early diagnosis and treatment of severe LDL-C deficiency. For both ABL and HHBL, treatment is typically initiated based on the clinical diagnosis, and centres on dietary modification and a strict adherence to high dose lipid-soluble vitamin replacement to prevent neurologic and ophthalmologic sequelae. Fat restriction to < 30% of total calories in the context of overall adequate caloric intake and avoidance of long-chain fatty acids generally improves gastrointestinal symptoms. High dose oral supplementation with vitamins A (100-400 IU/kg/day), E (100-300 IU/kg/day), D (800-1200 IU/day), and K (5-35 mg/week) are effective in preventing or even reversing complications, relying on the minimal residual lipoprotein function and alternative mechanisms for absorption, such as the medium chain TG pathway through the hepatic portal vein.

Past reports of ABL and homozygous FHBL patients have shown that the severity of neuropathy and retinopathy is related to the age at diagnosis and treatment, rather than to the nature of the mutation [7]. Vitamin E is particularly critical and effective in preventing progression of neurological sequelae, although serum levels often do not normalize even with high dose supplementation [14, 15]. Parenteral therapy offers no benefit above oral therapy, and in fact may increase the risk of hepatic steatosis. The early initiation of high dose lipid-soluble vitamin replacement and dietary modification in this patient likely prevented many of the devastating effects of severe hypobetalipoproteinemia, allowing him to be relatively free from neurological or visual impairment.

In the era of next generation DNA sequencing, distinguishing clinically significant mutations from harmless variants is a significant challenge, even with multiple in silico methods of predicting deleterious effects [16]. Careful genetic and clinical analysis of family members were crucial to elucidating the cause of his phenotype. This case demonstrates both the power of next-generation sequencing to obtain an accurate diagnosis in an extremely atypical case, as well as its potential to generate hypotheses when numerous rare variants are revealed in a patient’s genome.

## Conclusion

ABL and homozygous FHBL are rare genetic disorders characterized by severe hypolipidemia and fat soluble vitamin deficiency. Many of the disorders’ devastating sequelae are preventable with high dose oral vitamin supplementation and diet modification. Next-generation sequencing of the proband and family members were crucial in securing an accurate diagnosis of a patient with a classic ABL or homozygous FHBL phenotype, who did not have mutations clearly definitive for either discrete disorder. Compound heterozygous FHBL has so far been very rarely encountered, but may be a consideration in atypical cases. As the sophistication of genetic sequencing techniques improve, “genetic mysteries” like the present case may reveal greater genetic heterogeneity underlying these classic and rare disorders. At the same time, such advancements may often provide more new questions than answers, requiring ever more advanced bioinformatics and experimental data to keep pace.
